# Left Ventricular Apical Cannulation in Acute Type A Aortic Dissection

**DOI:** 10.3390/jcdd12110451

**Published:** 2025-11-19

**Authors:** Benedetto Ferraresi, Antonio Nenna, Mohamad Jawabra, Diletta Corrado, Filippo Barberi, Carmelo Dominici, Giovanni Casali, Massimo Chello, Mario Lusini

**Affiliations:** 1 Cardiac Surgery, Fondazione Policlinico Universitario Campus Bio-Medico, Via Alvaro del Portillo 200, 00128 Rome, Italy; benedetto.ferraresi@unicampus.it (B.F.); mohamad.jawabra@unicampus.it (M.J.); diletta.corrado@unicampus.it (D.C.); f.barberi@policlinicocampus.it (F.B.); m.chello@policlinicocampus.it (M.C.); m.lusini@policlinicocampus.it (M.L.); 2 Cardiac Surgery, Azienda Ospedaliero Universitaria Maggiore della Carità di Novara, Corso Mazzini 18, 28100 Novara, Italy

**Keywords:** aortic dissection, transcatheter, complication, emergency, cannulation

## Abstract

Background and objectives: In cases of acute type A aortic dissection, including iatrogenic cases following transcatheter procedures, the choice of arterial cannulation site has a critical influence on early haemodynamics, organ protection and the risk of malperfusion. Transapical left ventricular cannulation has been suggested as a ‘central’ approach for rapidly establishing cardiopulmonary bypass with antegrade true-lumen flow. This review summarises the current evidence on TAC in acute type A dissection, focusing on indications, technical aspects and clinical outcomes. Materials and methods: We conducted a narrative review of observational studies and technical reports describing TAC for the surgical repair of acute type A aortic dissection. Particular attention was paid to patient selection, operative technique, perioperative complications, and early and mid-term results. Results: Across the published series, TAC is primarily employed in haemodynamically unstable patients or when the peripheral arteries are dissected, diseased, or unsuitable. A long arterial cannula is introduced through the left ventricular apex, crosses the aortic valve and is positioned in the true lumen of the ascending aorta under echocardiographic guidance. This configuration enables the rapid initiation of CPB, shortens skin-to-pump times, and provides reliable antegrade inflow. Early mortality and stroke rates are comparable to those associated with other cannulation strategies. Reported complications include malperfusion requiring site conversion, apical bleeding and rare local structural damage. These can be minimised through standardised technique and systematic imaging. Conclusions: TAC is a valuable bail-out option and, in selected patients, a primary cannulation option for acute type A aortic dissection when conventional arterial access is unsafe or ineffective. Although it offers fast and reproducible establishment of antegrade true-lumen flow, it requires specific expertise in apical exposure and intraoperative echocardiography. It should therefore be integrated into a structured perfusion and repair strategy.

## 1. Introduction

The surgical management of acute type A aortic dissection (ATAAD) remains highly challenging, with significant perioperative mortality despite technical and organisational advances. In a ‘salvage’ (i.e., non-emergency) setting, such as in the case of iatrogenic aortic dissection following transcatheter procedures, the choice of arterial cannulation site for establishing extracorporeal circulation (ECC) is a widely debated topic, as it affects haemodynamics, organ protection, and the risk of complications [1,2]. Transapical cannulation has attracted attention as a “central” option, primarily due to its ability to ensure antegrade aortic flow and the speed with which it can be performed [3,4,5,6,7,8,9]. This makes it suitable for emergency situations involving haemodynamically unstable patients or when other sites are not feasible. Inserting a long cannula from the apex of the left ventricle through the aortic valve, preferably under echocardiographic guidance, can initiate cardiopulmonary bypass (CPB) rapidly and provide effective flow [3,10]. This could potentially reduce malperfusion phenomena related to false lumen filling [3,4]. However, there is no consensus for the procedural details, which remain highly heterogeneous [1,11]. In this context, apical cannulation is primarily documented in observational case series with inconsistent indications and outcomes. The aim of this review is to provide a critical summary, integrating selection criteria, technical aspects, and the impact on outcomes, based on the best available evidence and current practice [3,4,5,7,9].

## 2. Transapical/Left Ventricular Apex Cannulation: Similar Results, Different Procedural Aspects

The literature on transapical cannulation techniques reveals significant variations in all key steps. These include the access method (scalpel incision versus hemostatic forceps puncture), apex management (with or without purse-string sutures and felt pads), cannula calibre and type (e.g., 7 mm with mandrel versus 28 F reinforced), target positioning (cusps of the sinuses of Valsalva versus sinus-tubular junction) and imaging guidance and confirmation methods [3,4,5,10,12,13]. The indications and contraindications also vary, including the choice of rescue/salvage in cases where peripheral access fails or is contraindicated (e.g., femoral/axiallary dissection, iliofemoral disease or limb ischemia) [4,5,12]. All approaches aim to safely perfuse the antegrade circulation by introducing the cannula through the apex of the left ventricle, crossing the aortic valve, and positioning its tip in the true lumen of the ascending aorta under echocardiographic guidance. Crucial attention must be paid to hemostasis and cannula stability [3,4,6].

Djukanovic et al. elevated the heart and applied two purse-string sutures with felt pads around the left ventricular apex [1]. They made a 1 cm incision in the apex and inserted the cannula under TEE guidance. They then positioned the cannula tip through the aortic valve at the level of the sinuses of Valsalva. General contraindications for femoral artery cannulation included femoral artery dissection, iliofemoral disease, and limb ischaemia. The axillary artery was not considered suitable for cannulation when a large dissection flap extended beyond this artery [1].

After elevating the ventricular apex, Flege et al. made an incision in the epicardium near the apex and used the tip of a hemostat to penetrate the ventricular wall without cutting it [14]. The cannula was then inserted and guided through the aortic valve. A purse-string suture was used to prevent the cannula from slipping [14]. Transapical cannulation was performed in patients whose femoral and brachiocephalic arteries had been dissected, or in the absence of femoral pulses.

Fukuda et al. performed transapical cannulation following the failure of axillary artery perfusion due to a sudden increase in flow resistance [12]. The cardiac apex was elevated and a 3-0 polypropylene mattress suture with small felt pads was inserted. Then, a 28 F wire-reinforced venous cannula was inserted into the ascending aorta through the left ventricle [12].

Matsushita et al. made a 1 cm incision at the apex of the left ventricle and passed a 7 mm cannula with a stylet through the apex and aortic valve, using transoesophageal echocardiography to guide its position in the ascending aorta [5]. Transapical cannulation was chosen for cases involving a distal aortic aneurysm, iliofemoral disease, femoral artery dissection, or limb ischemia [5].

Shimamura et al. made a 1 cm incision at the apex of the left ventricle without using a purse-string suture to enable transapical cannulation [13]. Under transoesophageal echocardiographic guidance, the tip of the cannula was positioned in the true lumen of the ascending aorta [13].

Sosnowski et al. place a purse string in the right atrial appendage string in the right atrial appendage using 3-0 Tycron sutures [15]. A two-stage cannula is then inserted for venous drainage and connected in readiness for cardiopulmonary bypass. The technique has been used successfully in patients where the dissection flap involved the subclavian and femoral vessels [15].

Suenaga et al. used transapical cannulation in all patients except in cases where there was a contraindication, such as severe aortic stenosis or redo surgery [4]. They made a 1 cm incision in the apex of the left ventricle and passed a 7 mm cannula with a stylet through the apex and across the aortic valve, positioning it in the ascending aorta in the ascending aorta at the level of the sinotubular junction, under transoesophageal echocardiographic guidance [4].

After performing CT scans, Wada et al. performed transapical cannulation in patients for whom direct cannulation of the ascending aorta was difficult due to the inability to puncture the true lumen [6]. They made a 1 cm incision in the left ventricular cardiac apex with a scalpel, without using felt pads, and inserted a 7 mm cannula into the ascending aorta. Epiaortic ultrasonography and transoesophageal echocardiography were then used to confirm the cannula’s position and perfusion [6].

## 3. Trans-Apical Cannulation: An Optimal Bail-Out Strategy, but Requiring More Intense Surgical Scenario

In the decision-making process for acute type A aortic dissection, whether spontaneous or iatrogenic, the choice of cannulation site is crucial, as it determines the quality and direction of blood flow during the first few minutes of extracorporeal circulation.

A cannula inserted through the apex provides quick access to CPB and cooling, ensuring anterograde flow in the true lumen while preparing for hypothermic arrest. Once the distal anastomosis has been completed under circulatory arrest, the inflow is transferred to the graft, usually via an 8–10 mm side branch. At this stage, the prosthetic tube is deaerated and the apical cannula is removed before the apex is closed. CPB is then resumed from the side arm of the prosthesis to allow for rewarming and proximal/valvular repair [3,4,6,10,15].

There are two reasons why changing sites is necessary. Firstly, it frees the valve and root; a transvalvular cannula can induce or worsen aortic insufficiency and interfere with proximal manoeuvres. Retracting or removing the cannula eliminates these problems and makes valve repair or proximal aortic replacement easier. Secondly, changing sites stabilises perfusion and minimises the risk of malperfusion caused by changes in flow or tip displacement. This planned (post-distal) change must be distinguished from the ‘rescue’ change that is sometimes required earlier due to signs of malperfusion or new aortic regurgitation at the start of CPB. A review of the literature reveals that transapical cannulation is a rapid and reliable method of establishing antegrade flow in the true lumen, independent of peripheral vessels which are often diseased or affected by dissection. This approach has two clinically relevant advantages: it reduces both skin-to-pump time and the risk of iatrogenic malperfusion related to retroperfusion [4,5,7,10,16].

### 3.1. Advantages

The main advantages of apical cannulation over other methods are the speed and consistency of the blood flow. In time-critical situations such as acute type A dissection, entering CPB ‘in a matter of seconds’ can be crucial. Insertion is very rapid because peripheral dissection is not required. In Wada’s extensive case series, there were no intraoperative malperfusion, with a stroke rate of 5.8% [6]. These observations are reflected in direct comparisons. Suenaga found that skin-to-CEC time was halved compared to femoral time (23 ± 5.1 vs. 45 ± 16 min), with the same major outcomes [2,3,4,6,12].

A second advantage is pathophysiological: the cannula crosses the valve and is positioned in the true lumen of the ascending aorta. This provides antegrade flow and reduces the theoretical risks of false lumen perfusion and embolisation associated with femoral retroperfusion.

In practice, trans-apical cannulation (TAC) overcomes the limitations and downtime associated with peripheral approaches. It eliminates the need for a second incision or preparation of a small-calibre artery, which carries the risk of inadequate blood flow or iatrogenic dissection. Furthermore, there are no issues with limb ischemia. TAC has also proven to be a real lifesaver when peripheral routes ‘do not hold’, as in Fukuda’s case, where flow resistance in the axillary artery necessitated an immediate conversion to the apex, effectively restoring perfusion [2,12].

### 3.2. Disadvantages

However, the most worrying of the complications is malperfusion triggered by the start of CPB, even with antegrade flow from the apex; this is related to issues in cannula placement or kinking at the entrance site in the left ventricle of in the CPB circuit. In Matsushita’s series, apical cannulation required a change of site in five out of 52 patients: four cases were due to documented malperfusion and one case was due to worsening aortic insufficiency [5]. In all cases, switching to subclavian or femoral cannulation was required to correct the issue, and no deaths related to the change in cannulation were recorded. The proposed algorithm is pragmatic: bilateral radial monitoring and continuous transesophageal echocardiography (TEE). If signs of malperfusion appear, CPB is interrupted immediately and the circuit is reconstituted from another site. In other words, TAC does not eliminate the risk of malperfusion; however, when it occurs, it must be recognized and treated decisively [16].

Bleeding from the apex is the most common mechanical complication. Wada reports that three patients required additional sutures for haemostasis using a purse-string suture in the early stages. [3]. After standardizing a stab puncture without a purse-string suture and closure with 4-0 sutures on felt pledgets, no further bleeding requiring additional sutures was observed [17]. Although rare, apical fracture is a possible event in fragile or ischemic walls, or in cases of repeated manipulation. The precautions described by some authors reduce the risk.

In terms of neurologic events, TAC shows stroke rates that are similar to or sometimes lower than those of other strategies. In Wada’s historical case series, the clinical stroke rate was 5.8%, which was not attributed to cannulation [6]. Many of the deaths were related to preoperative malperfusion rather than inflow problems. Subsequent series report stroke rates of ~6–10%, with no significant difference compared to femoral or axillary access. This emphasizes that the determining factor is often the pathophysiology of the dissection, rather than the CPB entry site. Late local events at the apex are rare. Sosnowski did not observe pseudoaneurysms at the cannulation site with systematic CT follow-up, and Shimamura cites peri-apical hematoma as possible but ‘very rare’ [13,15]. Ultrasonographically, left ventricular function appears to be preserved and coaptation of the flaps against the cannula wall may mitigate acute insufficiency, as has been observed intraoperatively.

Overall, the early outcomes are at least comparable to those of other inflow strategies. In Suenaga’s comparative series, 30-day mortality was 4.3% with the transapical approach versus 8.8% with the femoral approach (non-significant difference), and the respective rates of post-operative stroke were 11% and 17% (non-significant difference) [4]. The main practical advantage was the shorter skin-to-ECC time (23 ± 5.1 versus 45 ± 16 min). Three- and five-year survival rates were similar between the groups (77.4% and 71.9%, respectively, for the transapical approach).

In Djukanovic’s study (*n* = 111), mortality was 16.7% in the transapical group versus 18.2% at other sites [1]. There were no differences in stroke, myocardial infarction or acute renal failure. In conclusion, there were comparable major outcomes and the CPB was established rapidly and safely [1].

Larger single-centre cohorts confirm an acceptable safety profile. Wada (*n* = 138) reports no intraoperative malperfusion events or site conversions, with consistently adequate flow [6]. Clinical stroke occurs in 5.8% of cases and in-hospital mortality is 18.8%, which is attributed to critical preoperative conditions rather than the technique. In the Matsushita series, the hospital mortality rate was 7.7%, and the stroke rate was 9.6%. CPB was completed via the apex in 90.4% of cases [5]. Conversion was necessary in 9.6% of cases (due to malperfusion at start-up in four cases and worsening of AI in one case). There were no deaths related to conversion. The reported rates of neurological events (6–14%, depending on cohorts and risk mix) are in line with other cannulations in the same era and population. Medium-term outcomes are favorable when the “distal open + graft perfusion” algorithm is adopted. In Shimamura’s series, the rate of freedom from distal reintervention was 95.9% at one year and 80.0% at five years [13]. When elective reintervention was necessary, the mortality rate was very low (3.0%), suggesting that the ‘hemiarch today, deferred distal treatment’ strategy is safe. Suenaga also demonstrates comparable five-year survival rates for transapical and femoral approaches, confirming non-inferiority in terms of duration [4].

As for the results, the available data indicate non-inferiority compared to other sites: in Djukanovic’s cohort, the major outcomes (mortality, stroke, kidney failure, rebleeding) are comparable between TAC and other sites, while in Suenaga’s cohort, TA was faster without neurological or medium-term survival penalties [1,4]. Wada’s extensive experience reinforces the idea of a “simple, fast, and reliable” technique on the true lumen. In summary: same safety, faster speed [3]. Complications of TAC are summarized in Table 1.

### 3.3. Safe Antegrade Inflow: Why the Apical Approach Often Prevails

Other approaches are well known and summarized in Table 2. Axillary artery cannulation has become increasingly popular and is often considered to provide ‘antegrade’ perfusion. From a haemodynamic standpoint, however, the situation is more nuanced. Although the inflow is more proximal than with femoral cannulation, flow to the aortic arch and descending aorta remains predominantly retrograde, with only a modest shift in the origin of the jet. Although axillary cannulation facilitates selective antegrade cerebral perfusion and avoids groin complications, it does not guarantee true-lumen perfusion in extensive dissections and should not be considered a ‘magic solution’ to malperfusion.

The carotid approach is an excellent alternative when available, offering anterograde flow to the brain with a relatively quick setup. However, it is not always ‘dissection-free’ and requires further exposure [3,4,7,8]. Double arterial cannulation (DAC) helps stabilise critical perfusion (e.g., by combining antegrade and retrograde flows to ‘push’ blood into compromised areas), but it adds complexity to the circuit and increases the number of sites at risk of complications. It is appropriate when, despite reasonable initial flow, organ perfusion remains poor [18]. When the true lumen of the ascending aorta is easily identifiable and accessible, both direct and transapical cannulation allow antegrade perfusion into the true lumen. In severely compromised or atheromatous anatomies, the transapical route is often the most ‘neutral’ option for quickly establishing reliable flow without manipulating the fragile wall. Direct cannulation of the ascending aorta avoids transvalvular passage but requires puncture of a dissected segment: if the true lumen is difficult to reach or the wall is very fragile, the risk of entering the false lumen increases if ultrasound control is not impeccable. In these scenarios, the transapical approach tends to be more repeatable and standardizable. In any case, outcomes depend more on the patient’s risk profile (preoperative malperfusion, instability) than on the inflow site, as indicated by the large cohorts treated with a transapical approach and good medium-term results.

## 4. Practical Recommendations

Considering the heterogeneous indications and procedural details of the literature, we propose a standardized sequence for transapical cannulation to reduce operator variability. Following median sternotomy and the establishment of venous drainage, transapical access is achieved via a small ventriculotomy at the apex of the left ventricle. An arterial cannula is then advanced antegrade through the aortic valve into the ascending aorta. Real-time transoesophageal echocardiographic (TEE) guidance is used to confirm the correct intraluminal position within the true lumen [3,4,6,7].

The indications in emergent/salvage procedure are

- contraindications to the femoral artery (e.g., aneurysm, complex distal district, iliofemoral disease, femoral dissection or limb ischemia) [1,5,10];
- dissection of the supra-aortic trunks, which makes cannulation of the axillary artery unreliable or impossible [4,6,7];
- severe diffuse atherosclerosis of the aorta [10,12];
- the need to quickly establish CPB for patients who are hemodynamically unstable or in cardiogenic shock [3,4,6].

### Step-by-Step Operative Procedure

**Figure 1:**
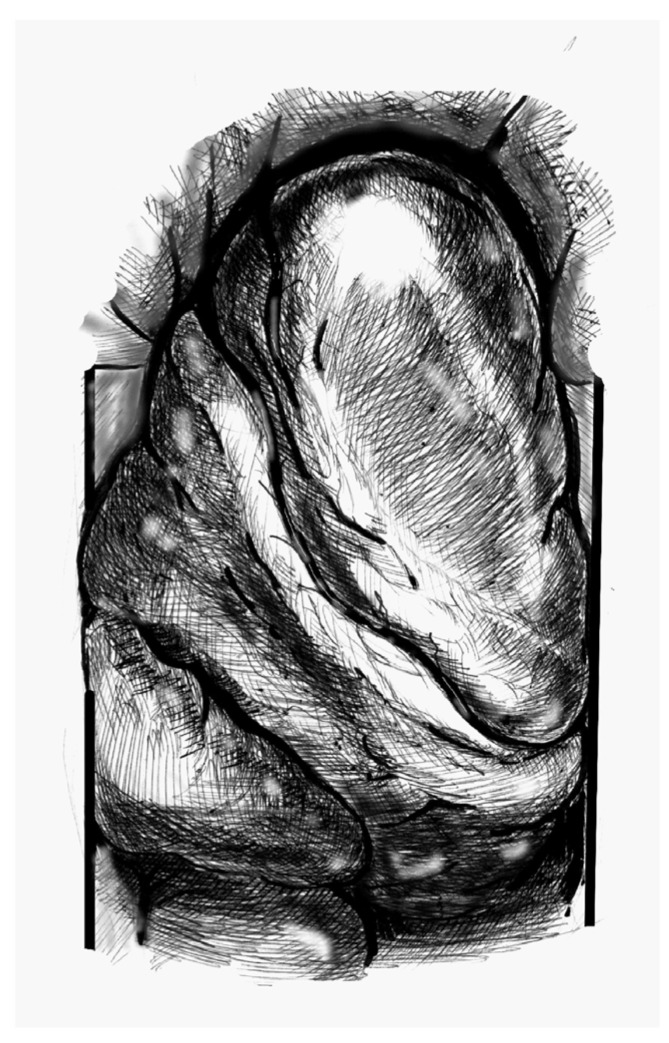
Heart positioning (Cardiac image viewed from the patient’s head).

**Figure 2:**
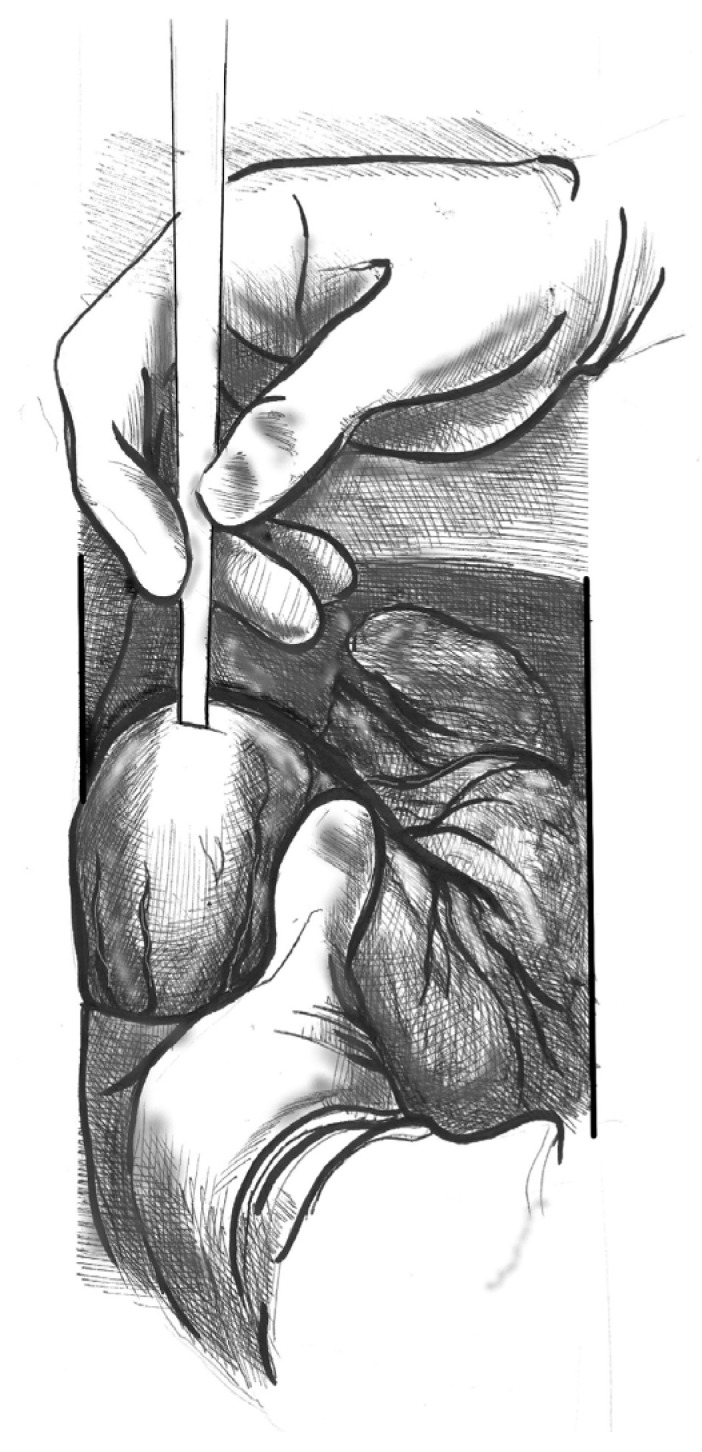
Blade incision in the anatomic apex.

**Figure 3:**
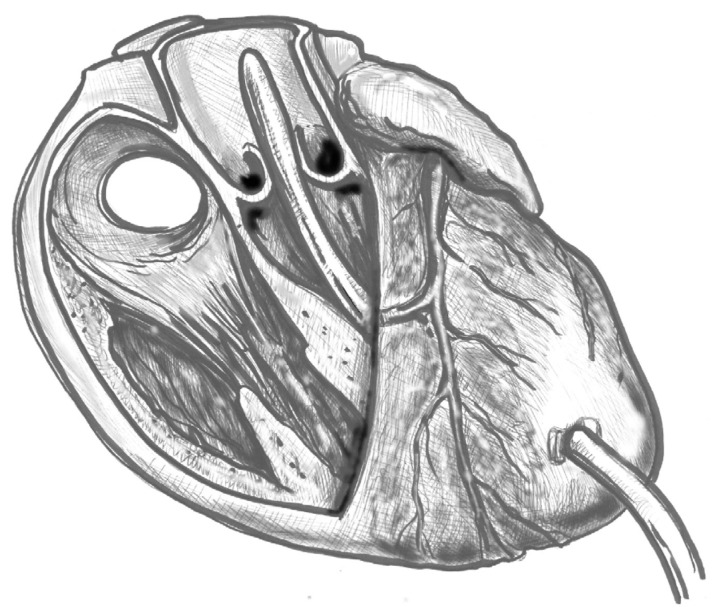
Cannula placement through the aortic valve.

**Figure 4:**
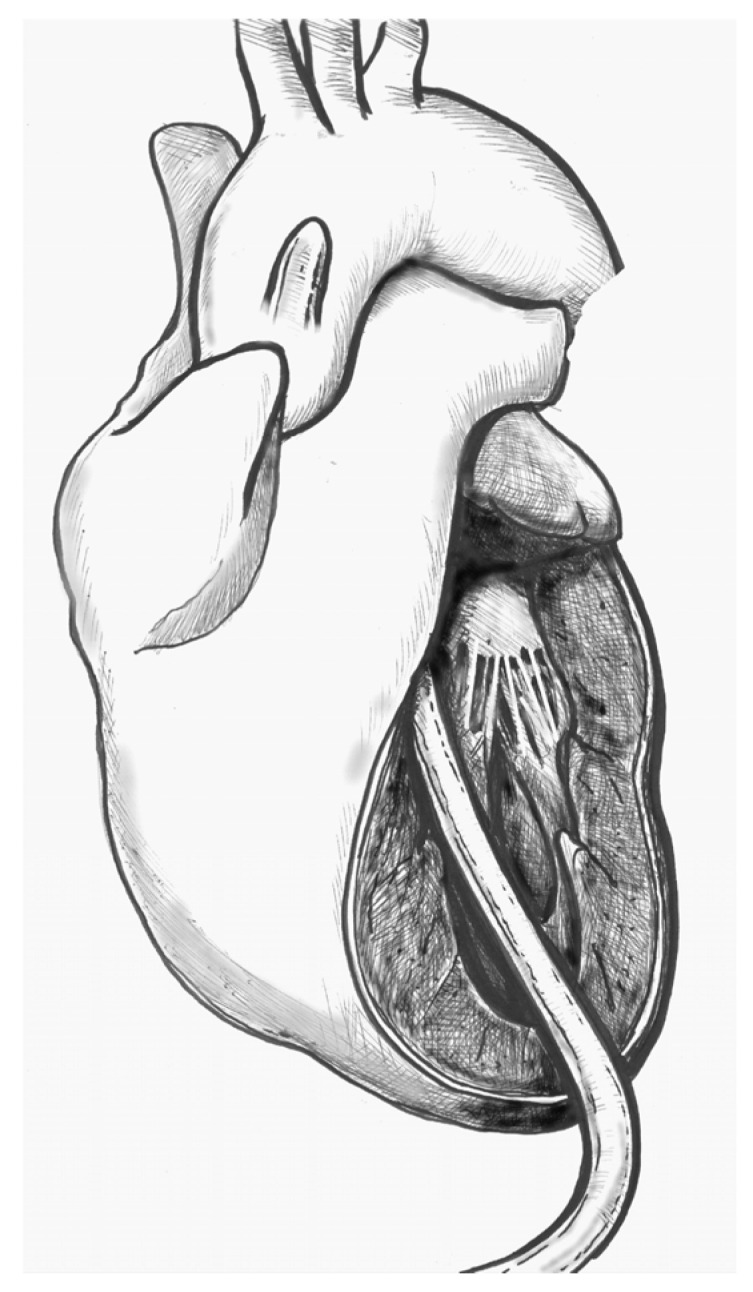
Final position of the cannula.

(1) Figure 1. Elevate the heart to expose the apex, then incise the epicardium. Maintain the trajectory towards the outflow tract/aortic valve to avoid deviating into the right ventricle (RV) or causing septal injury. Use transoesophageal echocardiography (TEE) at this stage to confirm the orientation.
(2) Figure 2. Make a small (approximately 1 cm) apical incision with a No. 11 blade. The incision should be oriented cranially towards the aortic valve. Insert the cannula through the apex.
(3) Figure 3. Gently advance the cannula along the LVOT axis towards the ascending aorta under continuous TEE guidance. Fix the cannula in place using two transmural prolene 2-0 sutures with pledgets and tourniquets.
(4) Figure 4. Ideally, the tip should pass the aortic valve and be positioned in the ascending aorta at the level of the sinus-tubular junction. Confirm this with TEE before starting CPB and reconfirm immediately after the start of perfusion. Avoid “short” positioning in the ventricular cavity, as this is ineffective and potentially dangerous.

## 5. Conclusions

Over the past two decades, transapical cannulation (TAC) has emerged as an effective method of rapidly entering CPB with reliable antegrade flow. It is particularly useful in cases of haemodynamic instability or when access is difficult due to distal aortic disease/aneurysm, iliac-femoral dissection/occlusion, limb ischaemia, or fragility/atheroma of the ascending aorta, all of which would make direct cannulation risky [3,6,10,12]. TAC enables CPB to be established quickly, with significantly shorter skin-to-CPB times and lower rates of mortality and neurological complications. However, TAC does not eliminate the risk of intraoperative malperfusion and requires expertise in intraoperative ultrasound (TEE/epiaortic) to confirm the true lumen. If the operator is inexperienced, relying solely on TEE can slow down the procedure. After distal anastomosis, it is also important to change the perfusion method: the apical cannula must be removed and reinserted into the graft to maintain antegrade perfusion and avoid leaving the transvalvular cannula in place for longer than necessary [1,4,7,8].

Put differently, this is not merely a “plan B” for challenging anatomies—it is a smart first step to buy time and gain control of perfusion. Immediate institution of CPB with reliable antegrade flow and early systemic cooling is best achieved by transapical cannulation, since this combination offers the best protection against cerebral and splanchnic malperfusion [3,4,7].

## Figures and Tables

**Table 1:** Complications of trans-apical cannulation in the current literature.

	In-Hospital Mortality	Postoperative Stroke	Myocardial Infarction	Acute Kidney Failure	Revision for Bleeding
Djukanovic, 2015 [1]	17%	6%	6%	9%	20%
Kusadokoro, 2020 [11]	18%	12%	-	5%	6%
Matsushita, 2012 [5]	8%	15%	-	17%	6%
Shimamura, 2018 [13]	8%	6%	3%	4%	5%
Suenaga, 2015 [4]	-	11%	-	2%	15%
Wada, 2006 [6]	19%	4%	5%	-	-

**Table 2:** Overview of cannulation strategies in acute type A aortic dissection.

Technique	Advantages	Disadvantages
Transapical	Very fast cardiopulmonary bypass start; antegrade true-lumen flow; no extra incisions; reliable when femoral/axillary are poor; easy to add second inflow.	Needs strict echocardiographic guidance and experience; not ideal with hard-to-cross valves; requires dedicated left ventricular vent; potential apical bleeding.
Femoral	Widely available, quick; useful as a bridge in arrest.	Retroperfusion leading to embolism and/or false-lumen malperfusion; limb ischemia; worsens with atheroma.
Axillary	More proximal inflow than femoral cannulation; facilitates selective antegrade cerebral perfusion; avoids groin complications.	Flow to the arch remains largely retrograde and true-lumen perfusion is not guaranteed in extensive dissections; slower; small/dissected vessels; local injury
Carotid/Innominate	Direct antegrade cerebral perfusion; rapid when anatomy is favorable.	Extra exposure; plaque/dissection risk; need contralateral protection.
Central aortic (direct)	Excellent when true lumen is secure; rapid central inflow.	Risk of false-lumen cannulation without rigorous ultrasound; manipulation of fragile wall.
Double arterial cannulation	Rescue when malperfusion appears/persists; allows pressure balancing.	Added complexity and lines; more potential complications; not inherently superior.

## Data Availability

No new data were created or analyzed in this study. Data sharing is not applicable to this article.
